# Aquatic Insects and Mycobacterium ulcerans: An Association Relevant to Buruli Ulcer Control?

**DOI:** 10.1371/journal.pmed.0040063

**Published:** 2007-02-27

**Authors:** Manuel T Silva, Françoise Portaels, Jorge Pedrosa

## Abstract

Texeira and colleagues discuss the association between arthropods and *M. ulcerans* in the light of a new study in *PLoS Medicine*.

Mycobacterium ulcerans infection, which can cause Buruli ulcer, is the third most common human mycobacteriosis worldwide, after tuberculosis and leprosy. Buruli ulcer occurs predominantly in humid tropical areas of Asia, Latin America, and, mainly, Africa, where the incidence has been increasing, surpassing tuberculosis and leprosy in some regions [1].

Buruli ulcer is a devastating, necrotizing, “skin-eating” disease of the poor, sometimes producing massive, disfiguring ulcers, with a huge social impact [1,2]. Furthermore, both Buruli ulcer and its pathogen have high scientific interest, with unique, enigmatic, and controversial features [1–4]. However, research on Buruli ulcer has been limited, although interest has grown since 1998, when the World Health Organization established the Global Buruli Ulcer Initiative, and in 2004 called for urgent action to control the disease and to increase research. There is no vaccine against Buruli ulcer and treatment remains difficult [1,2,5]. A detailed description of M. ulcerans infection, including its clinical aspects, is available at http://www.afip.org/Departments/infectious/bu/.

A new area of research is the association between arthropods and M. ulcerans. And now a new study in *PLoS Medicine*, by Marsollier and colleagues, takes our understanding of this association further.

## Arthropods and Mycobacteria

There is evidence that M. ulcerans is not transmitted person-to-person but is an environmental pathogen transmitted to humans from its aquatic niches [6,7]. However, it is not clear how this transmission occurs [6,7].

Arthropods can be vectors of many infectious agents. The hypothesis that arthropods were involved in the transmission of M. leprae to humans was originally put forward at the end of the 19th century [8]. This hypothesis was intermittently considered and tested until the early 1990s, but it was never consistently demonstrated.

**Figure 1:**
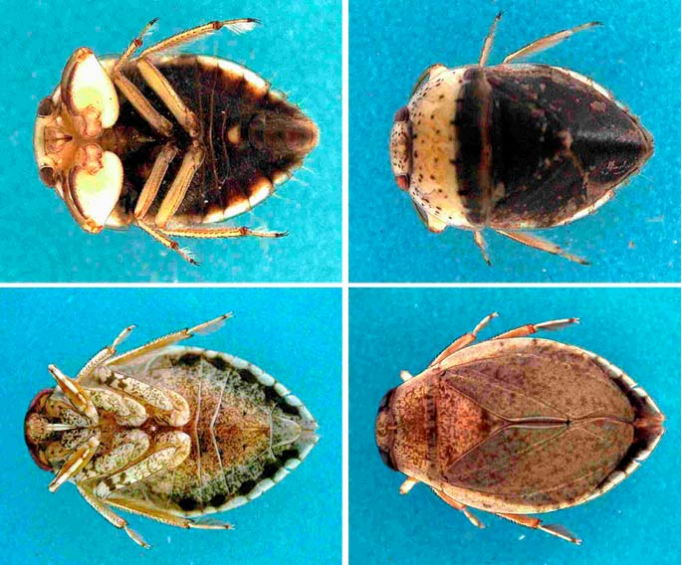
Semiaquatic Hemiptera That Have Tested Positive for M. ulcerans The top two images show Macrocoris sp. 1.0 cm in body length (family Naucoridae), and the bottom two images show Appasus sp., about 2.5 cm in body length (family Belostomatidae). The ventral and dorsal views are in the left and right photographs, respectively. Photo from [2].

The hypothesis that predatory aquatic insects, including those in the families Naucoridae and Belostomatidae (order Hemiptera) (Figure 1), were transmitters of M. ulcerans from aquatic niches to humans was advanced in 1999 [9]. The hypothesis was later reinforced by Marsollier and colleagues on the basis that [10]: (1) the salivary glands of Naucoris cimicoides are colonised with M. ulcerans upon feeding on grubs containing the pathogen; (2) M. ulcerans-infected N. cimicoides transmit the pathogen to mice upon biting; and (3) N. cimicoides in Buruli ulcer–endemic areas can be naturally colonised by M. ulcerans; this colonisation may occur through feeding on aquatic snails and fish, which take up M. ulcerans from water, mud, and aquatic plants [6,7].

These results have reawakened previous interest in the association between arthropods and human mycobacterioses, and have opened a new area in Buruli ulcer research.

## Immunity to Vector Antigens in Arthropod-Borne Diseases

Examples of arthropod-borne diseases are leishmaniasis and Lyme disease, transmitted by sand flies and by ticks, respectively. These haematophagous vectors bite the host's skin to take a blood meal. The bite introduces the pathogen along with saliva that profoundly alters the skin by molecules with antihemostatic activity (which enable the vector to take an effective meal) and immunosuppressive activity (which enhances the infectivity of the injected pathogen by counteracting the host immune response) [11–14]. Pre-exposure of mice to these salivary antigens induces protective immunity against pathogen transmission by neutralizing the immunosuppressive activity [11,12,14]. Furthermore, salivary molecules can adsorb to the pathogen [13,15]), a binding that can cause the microorganism to become an innocent bystander of the host's antisalivary immunity—again leading to protection against pathogen transmission [12].

Since efforts to develop vaccines targeting antigens of arthropod-borne pathogens have been largely disappointing, targeting arthropod salivary components provides a potential alternative mechanism to block the transmission of arthropod-borne diseases [11,12,14]. Indeed, vaccines have been described that protect mice against leishmaniasis by targeting sand fly salivary proteins [14].

The above results indicate that immunity to salivary antigens of vectors can be an epidemiological biomarker of insect biting and of the consequent immunoprotection against arthropod-borne diseases.

These observations prompted Marsollier and colleagues to search for immune signatures that could be correlates of protection against M. ulcerans and to investigate the relevance of vector salivary antigen–based vaccine strategies for Buruli ulcer.

## The New Study

Marsollier and colleagues' new study in *PLoS Medicine* [16] extends the authors' previous observations [10] and shows that repeated biting by M. ulcerans-free N. cimicoides renders mice more resistant to the infection obtained through biting by the insects carrying M. ulcerans. The researchers also found that subcutaneous immunization of mice with salivary extracts of M. ulcerans-free N. cimicoides protected against infection following injection of M. ulcerans, but only when the bacilli were first coated with salivary proteins. This suggests that the protection conferred to mice by previous N. cimicoides biting is associated with antibodies (detected in the blood of repeatedly bitten mice) reacting with proteins of N. cimicoides saliva that would bind to M. ulcerans during its stay in the insect's salivary glands, and would coat the bacilli when they are transmitted by biting.

The new study did not analyse the mechanism of this protection, but one possibility is that M. ulcerans transmitted by N. cimicoides to a sensitised host would become the innocent bystander target of the immune response against the adsorbed salivary proteins, resulting in host protection [12]. These results suggest that inhabitants of Buruli ulcer–endemic areas could become naturally immunised if repeatedly bitten by uninfected N. cimicoides, a mechanism for protection that has also been suggested for leishmaniasis [12,14,17] and Lyme disease [18]. The observation that unaffected, possibly resistant people exposed to aquatic environments in Buruli ulcer–endemic areas have higher titers of antibodies reacting with Naucoridae and Belostomatidae salivary proteins than do patients with Buruli ulcer in the same areas [16] is consistent with this hypothesis. This observation also suggests that biting of humans by N. cimicoides and immunity to those proteins occur in a natural setting.

## Limitations of the New Study

There are three major limitations to the new study. The first is that the study did not analyse whether the antibodies against insect salivary proteins in mice protected by prior biting by uninfected N. cimicoides are effectors of protection or only biomarkers of the protective status. Additional studies are, thus, necessary to clarify the roles of humoral and cell-mediated immunity in such protection.

Second, studies by Marsollier and others have been centered on Naucoridae, but it is possible that Belostomatidae and other predatory aquatic insects are also transmitters of M. ulcerans [9,16], which could complicate the investigation by extending the list of relevant insect salivary proteins. Another source of complication could be the occurrence of extensive polymorphisms in the relevant salivary proteins; the extent of these polymorphisms should be studied using specimens of aquatic insects collected in different geographical areas.

Third, a general lack of knowledge about the transmission of M. ulcerans is pertinent to this study—the overall relevance and contribution of biting by M. ulcerans-colonised aquatic insects to the transmission of Buruli ulcer is unknown. While sand flies and ticks are haematophagous, and biting is indispensable both for vector survival and for transmission of the disease [11], Naucoridae and Belostomatidae are carnivorous insects and only accidentally bite humans [7]. Therefore, other forms of transmission of M. ulcerans to humans, including skin trauma, have also been considered [1,5–7,19].

## Implications

If future work supports the notion that aquatic insects are important in the transmission of the causative organism in Buruli ulcer, as some data suggest [9,10,16], the results now published in *PLoS Medicine* could have important public health implications.

First, following the work with murine leishmaniasis [14], attempting to develop a Buruli ulcer vaccine that targets N. cimicoides salivary antigens would be justifiable. (It must be kept in mind, however, that the progress with vaccines against arthropod-borne diseases based on salivary proteins of vectors has been slow. Because of scientific, technical, and safety problems [12,14], such vaccines still remain unavailable for human use.) In addition, the presence of antibodies against salivary antigens of aquatic insects may be an important biomarker of protective status against Buruli ulcer, with epidemiological relevance in the study of populations at risk in endemic areas of the disease.
